# Editorial: Synaptic plasticity and dysfunction, friend or foe?

**DOI:** 10.3389/fnsyn.2023.1204605

**Published:** 2023-05-03

**Authors:** Fereshteh S. Nugent, Ka Wan Li, Lu Chen

**Affiliations:** ^1^F. Edward Hebert School of Medicine, Uniformed Services University, Bethesda, MD, United States; ^2^Department of Molecular and Cellular Neurobiology, Vrije Universiteit Amsterdam, Amsterdam, Netherlands; ^3^Departments of Neurosurgery, Neuropsychiatry and Behavioral Sciences, Stanford University School of Medicine, Palo Alto, CA, United States

**Keywords:** synaptic transmission, synaptic plasticity, early life adversity, long-term potentiation, long-term depression, metaplasticity, behavioral learning, Alzheimer's disease

## Introduction

Synaptic plasticity defined as the ability of neurons to modify their synaptic strength and connectivity as a function of activity, has long been postulated to mediate experience-dependent remodeling of neural circuits that ultimately underlies memory formation at various timescales. Since the discovery of hippocampal long-term potentiation (LTP), considerable progress has been made in our understanding of structural and mechanistic bases of different forms of synaptic plasticity that drive behavioral adaptation to the changing environment but also may confer our vulnerability or resilience to brain and behavioral pathology in response to adverse environmental factors, aging, and different types of trauma and insult across development (Südhof and Malenka, 2008; Nicoll, 2017; Li et al., 2019; Simmons et al., 2022). In this Research Topic, we highlight several conceptual advances in the field of synaptic plasticity that bridge the gaps between synaptic mechanisms underlying information processing in neuronal circuits of the healthy brain for normal behaviors. These articles provide insights into novel aspects of synaptic plasticity by linking newly identified molecular, synaptic and circuit correlates of synaptic structure, function, and behavioral learning. We also present two examples of dysregulation of synaptic plasticity as synaptic pathophysiological links to maladaptive circuit function that could underlie cognitive deficits and behavioral impairments related to psychiatric disorders using preclinical models of early life adversity and Alzheimer's disease.

## Papers in this collection

### Molecular signaling in synaptic structure and function

Our ability to adapt to the changing world replies largely on experience-dependent learning and memory formation, a process that requires synaptic plasticity. Synaptic plasticity is initiated by cascades of signal transduction, leading to synaptic structural reorganization and rearrangement of protein nano-machineries that changes synaptic efficacy and connectivity.

One of the main cellular mechanisms that control synaptic efficacy is the dynamic regulation of synaptic protein phosphorylation status by kinases and phosphatases (Feng and Zhang, 2009). Protein phosphatase-1 (PP1) is implicated in the changes of glutamatergic synapse activity and actin reorganization in dendritic spines, both of which are linked to the processes of neuroplasticity. The action of PP1 is regulated by a number of interactors, including neurabin (Munton et al., 2004). Foley et al. described an interesting finding that Inhibitor-2 positively regulates PP1 function in synaptic transmission, which is dictated by the threonine-72 phosphorylation on Inhibitor-2. Furthermore, using Förster resonance energy transfer /Fluorescence lifetime imaging microscopy studies, it was demonstrated that Inhibitor-2 enhances PP1γ interaction with its major synaptic scaffold, neurabin.

Structural plasticity of synapses correlates with changes in synaptic strength. For example, activation of NMDA receptors results in long-term enhancement of both dendritic spine size and synaptic strength (Herring and Nicoll, 2016). McLeod et al. here provide interesting evidence demonstrating that Wnt signaling promotes multi-innervated spines formation through neuronal nitric oxide synthase (nNOS)/NO/ soluble guanylate cyclase (sGC) signaling, leading to enhanced frequency and amplitude of excitatory postsynaptic currents. This finding provides an additional structural plasticity mechanism underlying LTP expression.

Dysfunction in synaptic proteins may lead to impairments in synaptic transmission or plasticity, thus impacting cognitive functions through altered neuronal circuit functions. Fragile X Syndrome (FXS) is a form of inherited intellectual disability caused by the loss-of-function mutations in the FMR1 gene. Key synaptic phenotypes in the FXS include exaggerated long-term synaptic depression (LTD) and impaired homeostatic synaptic plasticity, as well as altered spine density and morphology (Huber et al., 2002; Klemmer et al., 2011; Zhang et al., 2018). Gredell et al. showed that selective deletion of FMRP in a sparse subset of cortical layer 5 pyramidal neurons leads to altered structural dynamics of dendritic spines. Interestingly, although FMRP may operate cell-autonomously in this context during adolescence, additional non-cell-autonomous factors might also be involved in the regulation of synaptic phenotype in adults.

### Synaptic and circuit mechanisms underlying behavioral learning

The study by Romero-Barragán et al., examined the development of long-term synaptic plasticity at multiple hippocampal synaptic loci in response to high-frequency perforated path (PP) stimulation in the intact brain of behaving animals. They made the interesting observation that LTP can be induced not only at the ipsilateral PP-CA3 synapses where the presynaptic input received direct stimulation, but also at secondary downstream synapses such as CA3 to contralateral CA1 synapses, thus corroborating previous reports demonstrating polysynaptic “propagation” of LTP at synapses directly downstream of the stimulated ones (Buzsaki, 1988; Krug et al., 2001; Stepan et al., 2012; Taylor et al., 2016). Although the exact mechanism driving polysynaptic LTP induction is yet to be worked out, these studies provide an interesting perspective for studies investigating memory engram formation during behavioral learning.

In addition to LTP of excitatory synapses, inhibitory synaptic connections and their modification are known to be an integral component of circuit remodeling during behavioral learning. For example, in this collection of papers, Chen et al. showed that the inhibitory projections from the hippocampus to the medial septum bidirectionally control the speed of locomotion in mice, thus directly impacting exploratory behavior in mice. This unexpected role of hippocampal inhibitory output adds to the complexity of the hippocampus in cognitive functions.

Beyond the hippocampus, fear conditioning has been shown to induce long-term synaptic changes at both excitatory and inhibitory synapses in multiple brain regions, including the cerebellar cortex (Sacchetti et al., 2004; Scelfo et al., 2008). The study by Dubois and Liu investigated the inhibitory synapse function in the cerebellar cortex in the context of fear memory extinction. They showed that the enhanced spontaneous GABA release from cerebellar molecular layer interneurons after fear condition can be reversed by fear extinction, and that this reversal of learning-induced inhibitory synapse plasticity requires the GluN2D NMDA receptors. It is of note that the fear learning-induced enhancement of GABA release is not affected by GluN2D deletion, suggesting that different signaling pathways are at play in the induction and reversal processes of this form of synaptic plasticity. Reversal of long-term changes at excitatory synapse that occur during fear memory formation has been attributed to fear extinction [e.g., spine elimination and regrowth in the frontal association cortex during fear learning and extinction (Lai et al., 2012)]. Results from the study by Dubois and Liu further demonstrates the significance of inhibitory synapses plasticity in behavioral learning.

The developing and adult primary cortical areas are able to exhibit a form of widespread plasticity; i.e., cross-modal plasticity, that is triggered by the deprivation of input in one sensory modality (for example, deafness or blindness). The cross-modal plasticity increases the capabilities and performance of spared modalities in the affected individual that is dependent on the remaining senses in their everyday life (Bavelier and Neville, 2002; Ewall et al., 2021). In a mini review appearing in this collection, Lee describes the two components of adult cross-modal plasticity when a sensory loss results in cross-modal recruitment of the deprived primary sensory area for processing of the remaining senses as well as inducing a compensatory plasticity within the spared primary sensory cortices to enhance and refine the spared senses. She proposes the sliding threshold metaplasticity model as the mechanism that can account for synaptic plasticity related to both cross-modal recruitment and compensatory plasticity.

### Developmental-and aging-related synaptic dysfunction

Converging evidence from human and preclinical studies of early life stress/adversity (ELS/ELA) suggest that exposure to severe stress and adverse experiences during sensitive early developmental periods confer considerable risk for vulnerability to substance use disorder, depressive and anxiety phenotypes by triggering/altering synaptic plasticity in brain regions and neural circuits that are critical for cognitive functioning, mood regulation and motivated behavior (Lippard and Nemeroff, 2020; Simmons et al., 2022; Spadoni et al., 2022). In this collection, de Carvalho et al. used the limited bedding and nesting (LBN) model of ELA, which causes fragmented and unpredictable maternal care and neglect of pups (Molet et al., 2016). They found that LBN induced behavioral inflexibility in a reversal learning paradigm in both sexes, whereas LBN impaired goal-directed action strategies in male but not female mice. They also found sex-specific differences in the effects of LBN on synaptic transmission from cortical inputs to the dorsomedial or dorsolateral striatum ((DMS/DLS) where glutamatergic transmission was reduced in both DMS and DLS of male LBN mice while corticostriatal synaptic transmission was only affected in DMS of female LBN mice. Overall, this study provides sexually dimorphic synaptic and circuit mechanisms within the dorsal striatum with implications in ELA-induced impairments in goal directed behaviors.

Hippocampal LTP and LTD at Schaffer collateral-CA1 synapses can be elicited by activation of either NMDA or metabotropic glutamate (mGluR5) receptor activation (Palmer et al., 1997; Popkirov and Manahan-Vaughan, 2011; Wang et al., 2016). While the role of NMDA receptor-dependent hippocampal plasticity have been extensively studied for age- and Alzheimer's disease (AD)-related decline in cognitive functioning and learning and memory, less is known about the involvement of mGluR5-dependent hippocampal plasticity in this context. In this collection, Valdivia et al. used the APP/PS1 mouse model of AD (Lok et al., 2013) and the Chilean rodent model of natural AD (Octodon degus) (Tan et al., 2022) and found that while mGluR5-dependent plasticity was intact in young animals, it was lost with parallel cognitive deficits as animals aged. Given the conflicting result of a recent study demonstrating the potentiation of mGluR LTD in the APP/PS1 mouse model (Privitera et al., 2022), validation of loss of mGluR LTD in aging APP/PS1 mice and Octodon degus in this study is of interest for future investigations using preclinical AD models that exhibit natural age-related neurodegenerative processes common to the AD such as in Octodon degus AD model.

## Concluding remarks

The collection in this Research Topic serves as a vignette of the current efforts in the field of synaptic plasticity. These discoveries will continue to deepen our understanding of normal and pathological synaptic plasticity and we hope they fuel enthusiasm for future synaptic-based research on causal mechanistic links between structural and functional synaptic plasticity within brain circuits and networks influencing learning, reward and motivated behaviors in health and disease.

## Author contributions

FN, KL, and LC equally contributed to writing the article and approved the submitted version. All authors contributed to the article and approved the submitted version.
